# SF-36 Quality of Life Outcomes After Right Transradial Cerebral Angiography: A Prospective Short-Term Follow-Up Study

**DOI:** 10.3390/diagnostics16091292

**Published:** 2026-04-25

**Authors:** Johannes Rosskopf, Jens Dreyhaupt, Bernd Schmitz, Katharina Althaus

**Affiliations:** 1 Section of Neuroradiology, Bezirkskrankenhaus Guenzburg, 89312 Guenzburg, Germany; 2 Department of Diagnostic and Interventional Radiology, University Hospital Ulm, 89081 Ulm, Germany; 3 Institute of Epidemiology and Medical Biometry, University of Ulm, 89075 Ulm, Germany; 4 Department of Neurology, Christophsbad Medical Center, 73035 Goeppingen, Germany

**Keywords:** life of quality, transradial access, diagnostic cerebral angiography

## Abstract

**Background**: Quality of life (QoL) after transradial access in diagnostic cerebral angiography may be shaped by procedural demands as well as by the ambulatory setting itself. This study, for the first time, prospectively explored this dimension through follow-up assessments of QoL after the procedure. **Methods**: In this prospective study, QoL was assessed using the 36-Item Short Form Survey (SF-36), including the Physical and Mental Component Summary (PCS and MCS) as well as eight domain-specific subscales. After right transradial cerebral angiography, the SF-36 questionnaire was administered at baseline (pre-procedure), as well as at 1-month and 3-month follow-up visits. Mean PCS and MCS values were analyzed over time using linear mixed-effects regression models. In post hoc analyses, univariate and multivariable models were used to assess the influence of potential confounders. For subgroup analysis, patients were classified as transient deteriorators if PCS and/or MCS worsened by more than 0.5 SD at 1 month compared with baseline but not at 3 months. Permanent deteriorators were defined as worsening by more than 0.5 SD at both 1 month and 3 months compared with baseline. **Results**: A total of 35 patients (62.9% female) were recruited over the 12-month study period, with a mean age of 59.1 ± 10.1 years. No significant overall time effect was observed for mean PCS and MCS (*p* = 0.970 and *p* = 0.076). MCS showed a significant increase at 1 month compared with baseline (*p* = 0.046), with a trend toward significance at 3 months (*p* = 0.053). In post hoc analyses, sex, neurosurgical status, and dose area product were associated with MCS in univariate analyses (*p* < 0.05), but these associations did not persist after multivariable adjustment. For PCS, only age showed a significant association in univariate analysis (*p* < 0.05). In subgroup analyses, transient deterioration was more frequent in PCS than in MCS (11.4% [95% CI 3.2–26.7%] vs. 5.7% [95% CI 0.7–19.2%]), and permanent deterioration was also more common in PCS at 1- and 3-month follow-up (14.3% [95% CI 4.8–30.3%] vs. 8.6% [95% CI 1.8–23.1%]). Impairment predominantly involved the bodily pain subscale (88.9% [95% CI 51.8–99.7%]) within PCS and the vitality (80.0% [95% CI 28.4–99.5%]) and mental health sub-scales (80.0% [95% CI 28.4–99.5%]) within MCS. **Conclusions**: This short-term follow-up assessment demonstrated preserved QoL following transradial diagnostic cerebral angiography. Transient or permanent deterioration occurred in no more than five patients per subgroup (14%). These findings support the notion that a radial-first approach can be safely considered for diagnostic cerebral angiography without compromising patient-reported outcomes.

## 1. Introduction

Diagnostic cerebral catheter angiography continues to occupy a central position in the evaluation of a broad range of neurovascular disorders [1,2,3]. Despite major advances in non-invasive imaging technologies such as computed tomography angiography and magnetic resonance angiography, conventional digital subtraction angiography remains indispensable in many clinical scenarios. It provides unmatched spatial and temporal resolution and serves as the reference standard for the assessment of complex vascular lesions, including intracranial aneurysms, stenoses, arteriovenous malformations, and dural arteriovenous fistulas. In numerous indications, it retains a crucial role in pre-interventional planning, peri-procedural monitoring, and post-treatment surveillance [4,5].

In Neuroradiology, direct cervical carotid puncture was historically of primary importance but has recently seen a revival as a recognized alternative in patients with unfavorable arotic arch anatomy undergoing mechanical thrombectomy for acute ischemic stroke [6]. The transfemoral approach has long been the predominant access route in neuroradiological angiography [1]. It appears to be safe with a reported complication rate of 3.3%, even when performed in an ambulatory setting [2]. Nevertheless, the higher risk of potential puncture-site bleeding and ischemic complications remains the primary reason for prolonged postprocedural immobilization and in some cases overnight observation [7].

In Cardiology, the transradial access has for more than a decade been recommended as the first-choice arterial access in interventional procedures [8,9]. This recommendation is supported by several large randomized multicenter trials demonstrating consistent benefits in terms of safety and patient comfort compared with the transfemoral approach [9,10]. In recent years, the radial access has also been increasingly adopted for cerebral catheter angiography [11,12,13]. The radial access is associated with lower rates of puncture site complications and with consistent evidence for improved safety and patient comfort compared with the transfemoral approach [14,15,16,17,18]. In addition, it enables immediate postprocedural mobilization, which facilitates the transition from inpatient to outpatient care pathways and may, in turn, contribute to greater organizational efficiency and improved cost effectiveness [2,17,19]. Although its clinical advantages are well recognized and its use is steadily increasing, the transradial access has not yet become established as standard practice in cerebral angiography. Instead, its implementation remains heterogeneous and is still limited in many centers [20]. Although recent reports have identified potential risk factors for puncture-related complications after cerebrovascular angiography via the transradial approach like low body mass index [21] and even a single case report has described a mediastinal hematoma following transradial cerebral angiography [22], these observations alone do not fully account for the ongoing reluctance in clinical adoption. One possible reason might be the influence of the transradial access on health-related quality of life [20].

Health-related quality of Life (QoL) was recently found to be potentially affected after the transradial access in our own retrospective study [20]. In this former study, forty patients underwent diagnostic cerebral angiography, and QoL was assessed retrospectively using the SF-12 questionnaire. Applying the then-test approach, 12.5% of patients reported feeling worse after the procedure and this subgroup exhibited significantly lower physical and mental health scores. The transradial approach may exert a distinct influence on QoL in cerebral angiography due to procedural demands, patient awareness, and the predominantly ambulatory setting. Unlike procedures performed under general anesthesia, patients remain fully conscious during diagnostic cerebral angiography via radial access, which may heighten psychological stress in technically challenging situations [17,23]. Even minor postprocedural symptoms may affect perceived well-being, particularly when early discharge and rapid return to self-care are expected. Although the transradial approach is generally safe, transient vascular discomfort or temporary hand dysfunction may occur [24,25,26,27]. Taken together, these factors may subtly influence both the physical and mental dimensions of QoL.

QoL is commonly evaluated using standardized patient-reported outcome instruments that quantify different aspects of physical, psychological, and social functioning. Among these, the 36-Item Short Form Health Survey (SF-36) is one of the most established and comprehensive tools [28,29,30]. It covers eight health domains including physical functioning, role limitations due to physical health, bodily pain, general health perceptions, vitality, social functioning, role limitations due to emotional problems, and mental health which together allow a differentiated characterization of both physical and mental health status. In addition to the two composite indices, the Physical Component Summary (PCS) and the Mental Component Summary (MCS), the availability of separate domain scores enables the detection of subtle, domain-specific variations in functional capacity, perceived well-being, and psychosocial resilience.

In contrast, the shorter 12-Item Short Form (SF-12) represents a condensed derivative of the SF-36 in which several domains are aggregated into summary constructs [31,32]. While the SF-12 is efficient and well suited for large-scale surveys or routine clinical screening, its reduced item set offers less granularity, particularly with respect to role functioning, vitality, and social participation. As a consequence, more nuanced or domain-specific fluctuations, especially those occurring at the interface between physical strain, emotional stress, and everyday functioning, may remain undetected when relying solely on SF-12 composite scores [33].

The EQ-5D, by comparison, serves a different primary purpose. It is widely used in health-economic and policy-oriented contexts because it allows the derivation of utility values and quality-adjusted life years [34,35]. However, its structure, limited to five dimensions and a single self-rated health index, provides a more global rather than multidimensional representation of health status. While this makes the EQ-5D highly suitable for cost-effectiveness analyses, it offers considerably less sensitivity to small but clinically relevant changes in functional or psychosocial domains and is therefore less suited for detailed clinical research questions in which patient experience and functional adaptation are of central interest [36].

In longitudinal study designs with repeated measurements, instrument choice becomes particularly important, as potential intraindividual changes may occur gradually and vary across domains rather than presenting as marked global decline [37,38]. From a psychometric perspective, such settings require instruments with sufficient responsiveness, that is, the ability to detect small but clinically meaningful changes over time and to distinguish them from measurement variability [33,37]. Compared with the SF-12, the SF-36 offers greater measurement depth within each domain and therefore higher responsiveness, particularly in areas such as vitality, social functioning, and role limitations due to physical or emotional strain [37,39]. Its broader item coverage reduces the risk that subtle yet clinically relevant changes are absorbed into aggregated summary scores; a limitation that may arise more readily when using shorter instruments [39].

Moreover, repeated assessment across multiple SF-36 subscales permits the temporal evolution of different health dimensions to be distinguished, allowing researchers to determine, for example, whether changes primarily affect physical functioning, fatigue and vitality, or psychosocial adjustment [40]. Such domain-specific trajectory patterns are less accessible when relying solely on the SF-12, where several dimensions are condensed into two summary indices and nuanced longitudinal trends may therefore remain obscured [37]. In this context, the SF-36 provides a more informative and fine-grained profile for longitudinal analyses, enabling not only the detection of change, but also its localization within specific components of health-related QoL.

Therefore, the present study prospectively investigated health-related QoL after transradial diagnostic cerebral angiography using the SF-36. QoL was assessed at baseline prior to the intervention and again at one- and three-month follow-up in order to obtain a time-resolved perspective on patient-reported outcomes.

## 2. Materials and Methods

### 2.1. Study Design and Patient Selection

Since August 2020, the transradial access has been the primary access route for diagnostic cerebral catheter angiography at our institution. Our retrospective study evaluating the QoL outcomes of patients who underwent transradial angiography was conducted between January 2020 and March 2021 [20]. One year later, we initiated this prospective survey, which was carried out between March 2022 and February 2023. Inclusion criteria were age ≥ 18 years, non-pregnant status, cognitive ability to participate in the telephone follow-up, and willingness to take part in the follow-up assessments. Exclusion criteria were lack of written informed consent or absence of follow-up response; however, the latter did not occur in our cohort. Prior to the scheduled procedure, eligible patients received a postal invitation containing written study information and the SF-36 questionnaire. Patients who agreed to participate were asked to complete the SF-36 at home and bring the completed questionnaire with them on the day of their diagnostic cerebral angiography at our institution. These data were used as baseline QoL measurements. A standardized questionnaire was completed by the performing neuroradiologist immediately after each procedure. Follow-up interviews were conducted by telephone at 4 and 12 weeks after the procedure. The study cohort has been previously published [41]. However, analyses of the SF-36–based QoL data have not yet been reported. The study was conducted in accordance with the Declaration of Helsinki and approved by the Institutional Review Board of the University of Ulm (protocol code #420/21, approval date: 24 January 2022).

### 2.2. Transradial Approach

Proper positioning of the arm selected for transradial access is of critical importance during diagnostic cerebral angiography. In our experience, provisional arm support constructed from towels or cushions is not suitable for centers that routinely perform, or intend to routinely implement, transradial access. Instead, a stable and reproducible positioning system is essential to ensure operator comfort, procedural safety, and optimal catheter control. The following description refers rather exclusively to right-sided radial access, which represents our institutional standard approach. A brief note on left-sided radial access is provided at the end of the section; however, this approach was not required in any of the patients included in the present study.

**Figure 1:**
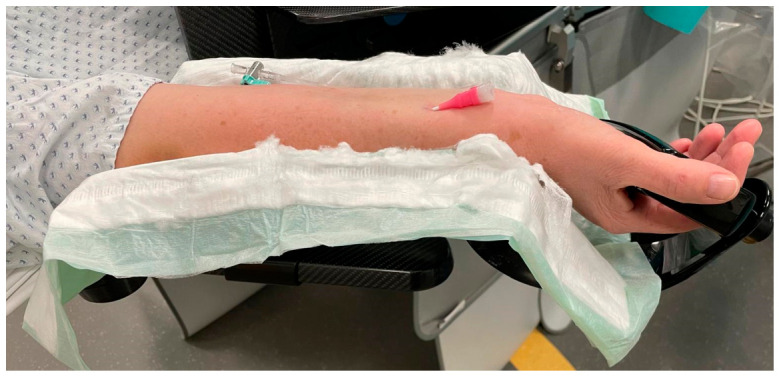
Right forearm in full supination, secured on a carbon angiography arm board for stable procedural positioning. The already placed 5-French Glidesheath Slender^®^ (Terumo Germany GmbH, Eschborn, Germany) is visible on the volar aspect of the wrist.

All procedures were performed with the patient in the supine position. The right forearm was positioned in full supination and securely fixed on a dedicated carbon angiography arm board to ensure stable working conditions throughout the intervention (Figure 1).

Radial artery access was obtained under sterile conditions. Approximately 10 mL of local anesthetic (Prilocaine hydrochloride 10 mg/mL; Xylonest^®^ 1%, Aspen Germany GmbH, Munich, Germany) was injected subcutaneously at the puncture site to minimize procedural pain and reduce the risk of radial artery vasospasm.

Neither routine ultrasound guidance for vascular access nor preprocedural assessment of collateral circulation (e.g., Allen’s or Barbeau test) was performed as part of the institutional standard protocol.

Radial puncture was performed by palpation using a 20-gauge needle, followed by insertion of a 5-French Glidesheath Slender^®^ (Terumo Germany GmbH, Eschborn, Germany) using the Seldinger technique. Following confirmation of correct sheath position using a DSA control run with contrast administration through the sheath, verapamil (2.5 mg) and heparin (5000 IU) were administered intra-arterially over 2–3 min to prevent vasospasm and thromboembolic events while minimizing systemic hypotensive effects.

Selective catheterization of the target vessels was performed using a 0.035″ guidewire and a diagnostic catheter, most commonly a 5-French Simmons-2 configuration. Continuous flushing of the diagnostic catheter with heparinized saline was maintained throughout the procedure to prevent thrombus formation.

**Figure 2:**
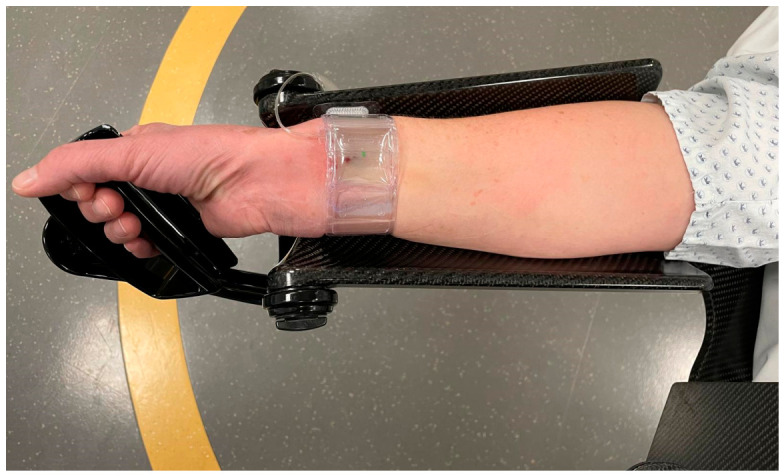
Hemostasis after the procedure was achieved using a TR BAND^®^ radial compression device (Terumo Germany GmbH, Eschborn, Germany).

Upon completion of angiography, hemostasis was achieved using a TR BAND^®^ radial compression device (Terumo Germany GmbH, Eschborn, Germany) (Figure 2). Gradual air titration was applied to ensure patent hemostasis, and the device was slowly deflated over approximately two hours before removal. Patients remained fully ambulatory during the compression period.

For left radial artery access via a volar approach, the angiography arm board is first positioned on the patient’s left side. The puncture is most safely performed using a mirrored technique relative to right radial access. In the supine position, the left arm is slightly abducted and placed on the arm board, and the operator positions her- or himself on the patient’s left side.

Radial artery puncture is performed by palpation, followed by insertion of the radial sheath using the Seldinger technique. After sheath placement, the forearm is repositioned and brought across the patient’s abdomen. Dedicated support elements stabilize the elbow dorsally to prevent inward rotation or return to the initial arm position.

For purely diagnostic angiographic procedures, distal radial access in the anatomical snuffbox is particularly advantageous on the left side, as, compared with the volar wrist puncture, it results in a less tortuous catheter course when the forearm is positioned across the abdomen.

Although the left radial artery may be considered in selected anatomical or procedural scenarios, a detailed description of left-sided radial positioning and access is beyond the scope of this report, as left radial access was not required in any patient included in this study.

### 2.3. Follow-Up Assessment by Structured Telephone Interview

The purpose of the interview was to evaluate health-related quality of life after angiography using the patient-reported SF-36 questionnaire. Follow-up telephone assessments were scheduled with all patients on the day of the intervention. The predefined follow-up time points were 1 month and 3 months after the procedure. A tolerance window of up to ±2 days around each time point (e.g., to account for weekends or public holidays) was defined a priori and considered acceptable for follow-up completion.

The SF-36 is a validated, generic instrument for measuring health-related QoL across a broad range of clinical and research contexts [28,42,43]. Compared with its abbreviated derivative (SF-12), the SF-36 provides a more detailed representation of patient-reported health status and functional impairment. This higher degree of granularity is particularly advantageous in longitudinal study designs, in which subtle temporal changes in physical and psychosocial functioning are of interest.

The instrument comprises eight domains: physical functioning, role-physical, bodily pain, general health, vitality, social functioning, role-emotional, and mental health. These domains may be analyzed individually and are additionally aggregated into two composite indices, the Physical Component Summary (PCS) and Mental Component Summary (MCS). All scores are norm-based and standardized to a population mean ± SD of 50 ± 10, expressed on a 0–100 scale. Lower scores indicate greater health limitations, whereas higher scores reflect better perceived health status [44,45].

In the present study, the SF-36 was administered as a patient-reported outcome measure within a structured telephone interview. The questionnaire was completed by trained research staff, who read each item verbatim from a standardized script and documented patient responses in real time.

SF-36 domain and summary scores were calculated in accordance with the standardized scoring algorithms provided by the instrument developers, including item recoding, z-transformation, and norm-based scoring procedures [28].

### 2.4. Statistical Analysis

All statistical analyses were performed using IBM SPSS Statistics (Version 30; IBM Corp., Armonk, NY, USA) and SAS, version 9.4 (The SAS Institute, Cary, NC, USA).

Demographic characteristics (age, sex, BMI) and procedural variables (angiography before/after endovascular treatment, radial access site, dose area product, total duration, and fluoroscopy time) were summarized using descriptive statistics. Continuous variables were reported as mean values with corresponding standard deviations (SD). Categorical variables were presented as absolute frequencies and percentages.

Boxplots were used to visualize the distribution of MCS and PCS scores at baseline and at 1 and 3 months follow-up. Individual trajectories were plotted to show the time course of MCS and PCS.

All analyses were conducted as a complete-case analysis, as no missing data occurred in the study cohort.

MCS and PCS scores were presented as means with corresponding SDs at baseline and at 1- and 3-month follow-up. Longitudinal trajectories of PCS and MCS were modeled using linear mixed-effects regression models including a subject-specific random effects to account for within-subject correlation of repeated measures.

In a two-step post hoc analysis, the potential confounders age, sex, neurosurgical status, BMI, DAP, total duration, and fluoroscopy time were included in the regression analysis. In the first step, univariate linear mixed-effects regression models were investigated for PCS and MCS. Multiple (multivariable) linear mixed-effects regression models were used in the second step for a simultaneously investigation of all significant confounder from the first step. A two-sided *p*-value < 0.05 was considered statistically significant. Due to the exploratory nature of this study, all results from statistical tests have to be interpreted as hypothesis-generating. An adjustment for multiple testing was not performed.

For subgroup analyses, clinically relevant deterioration in health-related QoL was defined a priori as a reduction of more than 0.5 SD from baseline in PCS or MCS scores. This threshold was chosen in accordance with the findings of Norman et al. [46], who demonstrated that, the minimal important difference in QoL measures approximates half a SD. Based on this criterion, patients were classified as transient deteriorators if they exhibited a more than 0.5 SD decline at 1 month but no longer met this threshold at 3 months. Permanent deteriorators were defined as those with a sustained more than 0.5 SD decline at both 1- and 3-month follow-up compared with baseline. Due to the small number of deteriorators, results were presented as graphical individual trajectories. The limited sample size within the respective subgroups precluded meaningful advanced association analyses.

The eight PCS and MCS subscales were analyzed in the pooled group of deteriorators. Within each component, a subscale was considered predominant if it showed a decline of more than 0.5 SDs at 1-month follow-up compared with baseline in the largest proportion of patients. For the PCS, the subscales Physical Functioning, Role Physical, bodily Pain, and General Health Perception were evaluated. For MCS, the subscales Role Emotional, vitality, mental health, and Social Functioning were assessed. If a comparable proportion of patients demonstrated deterioration in more than one subscale within a component, all respective subscales were reported as predominant.

## 3. Results

### 3.1. Study Sample

In total, 35 patients were prospectively enrolled during the 12-month study period (mean age 59.1 ± 10.1 years; 63% female). All procedures were performed via right radial artery access, with 86% representing follow-up examinations after prior endovascular or neurosurgical treatment. After sheath placement, digital subtraction angiography of the access site was routinely performed; no dissection or perforation was detected. Access-site complications were systematically documented by the treating neurointerventionalist immediately after the procedure; no relevant hematoma or vasospasm was observed. No patient required repeat digital subtraction angiography during the follow-up period. Only one patient was left-handed, while all others were right-handed. Most procedures (30/35, 85.7%) were conducted in a follow-up setting after prior endovascular or neurosurgical treatment, with all interventions performed via transfemoral access. Clinical and procedure-specific characteristics are summarized in Table 1.

### 3.2. Follow-Up Assessment of SF-36 Using Linear Mixed-Effects Models

**Figure 3:**
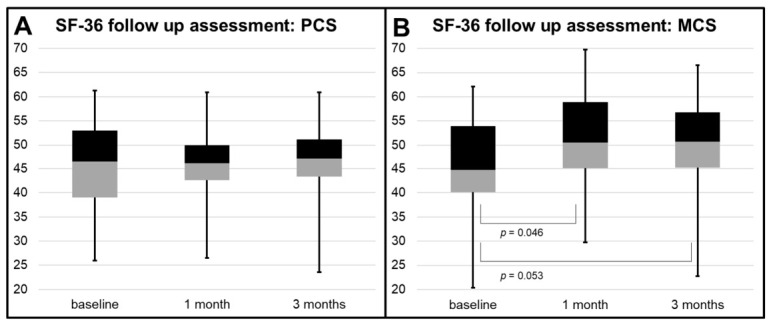
Longitudinal SF-36 assessment from baseline to 1- and 3-month follow-up. (**A**) Boxplots of the Physical Component Summary (PCS). (**B**) Boxplots of the Mental Component Summary (MCS). Mean MCS scores increased at 1 month compared with baseline (*p* = 0.046), with a trend toward significance at 3 months (*p* = 0.053). No significant changes over time were observed for PCS. Abbreviations: PCS, Physical Component Summary; MCS, Mental Component Summary.

No significant overall time effect was observed for mean PCS and MCS (*p* = 0.970 and *p* = 0.076, respectively). However, MCS increased significantly at 1 month compared with baseline (45.5 ± 10.6 vs. 50.2 ± 9.0; *p* = 0.046), with a trend toward significance at 3 months compared to baseline (50.1 ± 9.7; *p* = 0.053). In contrast, mean PCS remained stable over time, with no significant changes from baseline to 1-month (45.7 ± 9.0 vs. 45.9 ± 7.9; *p* = 0.908) or 3-month follow-up (46.2 ± 8.8; *p* = 0.806). The longitudinal courses of PCS and MCS are illustrated in Figure 3.

Post hoc analyses of MCS showed that sex, angiography before/after endovascular treatment, and DAP were significantly associated with MCS in univariate models, while fluoroscopy time showed a borderline association (Table 2). However, in the multivariable analysis adjusting for potential confounders, none of these variables remained statistically significant. Exclusion of fluoroscopy time from the multivariable model did not materially change these findings.

For the physical component score (PCS), only age was significantly associated with PCS in univariate analysis, whereas all other variables showed no significant associations. Therefore, no multivariable model was performed for PCS (Table 3).

#### Subgroup Analyses of Transient and Permanent Deteriorators

Transient deterioration was slightly more frequent in PCS than in MCS (11.4% [95% CI 3.2–26.7%] vs. 5.7% [95% CI 0.7–19.2%]). At the 1-month follow-up, PCS scores decreased by 4.8, 6.3, 6.4, and 6.8 points, while MCS scores decreased by 13.4 and 15.4 points compared with baseline.

**Figure 4:**
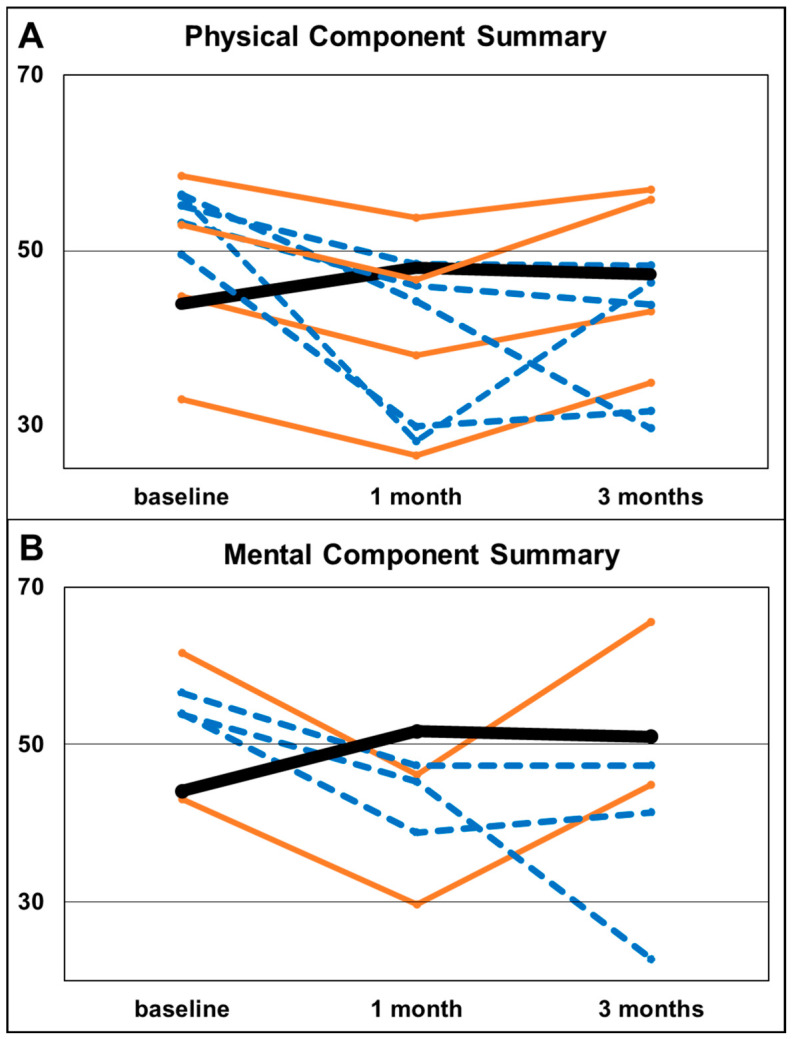
Individual trajectories divided into PCS (physical component summary), (**A**) and MCS (mental component summary), (**B**) showing transient deteriorators (orange line), permanent deteriorators (blue dashed line) and mean scores for the remaining patients (one black line). Transient deterioration was defined as a more than 0.5 SD decline at 1 month but not at 3 months compared with baseline; permanent deterioration as a more than 0.5 SD decline at both time points.

Permanent deterioration was also more common in PCS than in MCS at both 1- and 3-month follow-up (14.3% [95% CI 4.8–30.3%] vs. 8.6% [95% CI 1.8–23.1%]). PCS scores decreased in the five individual patients from baseline to 1 month (baseline to 3 months) by 12.1 (26.7), 6.7 (6.8), 28.0 (9.9), 7.2 (9.4), and 19.7 (17.9) points, respectively. In the three individual patients, MCS scores showed decreases of 8.6 (31.1), 9.2 (9.2), and 15.1 (12.5) points from baseline to 1 month (baseline to 3 months), respectively. Individual trajectories are illustrated in Figure 4. Mean (SD) scores for the remaining patients at baseline, 1 month, and 3 months were 44.1 (10.6), 51.7 (8.5), and 51.0 (8.5) for MCS and 43.9 (8.8), 47.9 (6.1), and 47.2 (8.4) for PCS.

Impairment predominantly involved the bodily pain subscale (89% [95% CI 51.8–99.7%]) within PCS and the vitality (80.0% [95% CI 28.4–99.5%]) and mental health subscales (80.0% [95% CI 28.4–99.5%]) within MCS (Table 4).

## 4. Discussion

Quality of life was prospectively assessed using the SF-36 questionnaire before transradial access for cerebral diagnostic catheter angiography and at 1- and 3-month follow-up. No significant overall time effect was observed for mean PCS or MCS. However, MCS increased significantly at 1 month compared with baseline, with a trend toward significance at 3 months. In a small subgroup of up to five patients (14%) transient or permanent worsening of QoL occurred, predominantly affecting bodily pain, vitality and mental health subscales.

In the present study, the prospective design enabled, for the first time in the neuroradiology literature, a longitudinal evaluation of health-related QoL scores with baseline data obtained prior to transradial access. At baseline, SF-36–derived MCS and PCS scores were comparable to previously reported values in patients with neurovascular diseases. Pala et al. [47] reported PCS and MCS scores of 43.7 and 45.1, respectively, after treatment of unruptured intracranial aneurysms in 79 patients. Similarly, Dammann et al. [48] reported PCS and MCS scores of 48.6 and 50.3 after treatment of unruptured aneurysms, while Dandurand et al. [49] found corresponding scores of 46.2 and 47.1. In our cohort, baseline PCS and MCS scores were similar, at 45.7 and 45.1, respectively.

While PCS scores did not show a statistically significant change over the observation period in the linear mixed-effects regression model analysis, MCS scores increased significantly between baseline and the 1-month follow-up, with a similar trend observed at the 3-month follow-up. To further explore potential factors influencing these findings, post hoc analyses were performed. In these analyses, several variables were associated with MCS in univariate models. However, these associations did not persist after multivariable adjustment, suggesting that the observed changes in MCS are not attributable to specific factors. For PCS, only age showed a significant association in univariate analysis, suggesting the absence of independent associations.

These findings are consistent with results reported in the cardiology literature. Cooper et al. [50] also observed improved QoL one week after a transradial procedure in 101 patients using the SF-36. Similarly, studies assessing QoL after transradial access have demonstrated improved QoL at follow-up visits, e.g., in patients with ST-segment elevation myocardial infarction [51,52,53]. One potential explanation for the observed postprocedural improvement may be the presence of response bias [54]. Patients may underreport persisting symptoms at follow-up evaluations, particularly when the overall outcome, such as successful treatment of an intracranial aneurysm, is perceived positively. Consistent with previous studies, we did not systematically assess patients’ subjective appraisal of the diagnostic procedure itself, even though the majority of patients in our cohort (86%) underwent angiography as part of routine follow-up after prior endovascular or neurosurgical treatment.

In the current study, subgroup analyses identified a relatively small number of patients with transient or permanent deterioration in QoL, affecting both PCS and MCS scores. These findings were limited to very few individual cases, ranging from two to five patients per subgroup. Similarly, in our previous retrospective study using the SF-12 questionnaire, we observed a small number of patients (12.5%) who rated their health status as worse following the procedure [20]. Roczniak et al. [55] reported that brachial access was associated with more frequent self-care difficulties compared with radial and femoral access after invasive cardiology procedures. In the present study, subscale analysis of the SF-36 revealed that impairment was most frequently observed in the bodily pain domain (89%), as well as in the vitality and mental health domains (80% each). The latter two domains may indicate an underlying psychological or mental strain, while the former could also be related to wrist deterioration, a potential complication previously described in association with transradial access [26,27,41,56].

An increasing number of technical and procedural investigations are contributing to the expanding body of evidence on transradial access in neuroradiology. Although our experience did not confirm this, ultrasound-guided puncture has been shown to decrease access failure rates [57] and is standard practice in many institutions. Alternative approaches include distal versus proximal radial access, ulnar access, and right- versus left-sided access routes [58,59,60,61,62]. In contrast, the benefit of heparin administration during transradial neuroangiography remains a matter of debate [63,64], despite its routine use in our institution. Pharmacological vasospasm prophylaxis, such as intra-arterial papaverine, a direct smooth muscle relaxant, is under investigation [65]. We consider routine vasospasm prophylaxis advisable, which may have contributed to the absence of vascular complications (0%) in our study population.

This study was not without limitations. First, the relatively small sample size limited the generalizability of the findings. However, given the exploratory nature of this study and its aim to investigate potential effects of transradial access on QoL, the sample size appears acceptable. To our knowledge, this is the first study in the field of neurointervention to address this question prospectively, and the present findings build upon and extend our previous retrospective investigations [20]. Second, the assessment of quality of life relied on patient-reported measures and may therefore be subject to response bias, an inherent limitation of questionnaire-based outcome evaluation. Moreover, the absence of staff blinding may have further influenced patient-reported outcomes through subtle interactions or expectations, thereby introducing an additional source of bias in the evaluation of quality of life. Third, although objective assessments such as duplex ultrasound might have allowed further correlation analyses, this modality is not routinely performed in our clinical practice. Importantly, digital subtraction angiography of the access site showed no vascular injury, and no crossover to transfemoral access occurred. Thus, the additional diagnostic value of routine ultrasound in this context is likely limited. Larger prospective studies incorporating objective assessments and comparisons with alternative approaches, such as transfemoral access, are warranted to further strengthen the evidence base. Such investigations would help to confirm and refine the present findings within a more robust methodological framework.

## 5. Conclusions

Over the 3-month follow-up period, health-related QoL remained stable after transradial diagnostic cerebral angiography, with no significant overall decline in PCS or MCS SF-36 scores. Transient or permanent worsening was observed in a small number of cases, involving no more than five patients per subgroup, and predominantly affecting the bodily pain, vitality, and mental health domains. Consequently, the present results lend further support to a radial-first strategy for diagnostic cerebral angiography.

## Figures and Tables

**Table 1:** Patients’ characteristics and procedural variables.

Variable	Total (*n* = 35)
**Age (years) ^1^**	59.1 ± 10.1
**Sex (f/m) ^2^**	22/13 (62.9%/37.1%)
**Angiography before/after endovascular treatment ^3^**	4/31
**Radial access site (right/left)**	35/0
**BMI (kg/m^2^) ^4^**	27.9 ± 5.3
**DAP (µGym^2^) ^5^**	3515.2 ± 2812.0
**Total duration (minutes) ^6^**	22.2 ± 11.7
**Fluoroscopy time (minutes) ^7^**	8.5 ± 4.6

^1^ Mean ± standard deviation. ^2^ female/male. ^3^ Number of patients before/after endovascular treatment. ^4^ Body mass index. ^5^ Dose area product. ^6^ Total duration of the procedure in the angiosuite. ^7^ Fluoroscopy time of the procedure in the angiosuite.

**Table 2:** Post hoc univariate and multivariable analysis of MCS adjusted for confounders.

Variable	Univariate Model β (SE)	*p*-Value	Multivariable Model β (SE)	*p*-Value	Multivariable Model β (SE) (Without Fluoroscopy Time)	*p*-Value
**Age**	0.006 (0.096)	0.948	–	–	–	–
**Sex**	4.608 (1.935)	**0.019**	2.394 (2.153)	0.269	2.412 (2.108)	0.255
**Angiography** **before/after** **endovascular treatment**	8.962 (2.886)	**0.003**	5.043 (3.902)	0.199	5.055 (3.873)	0.195
**BMI**	0.185 (0.182)	0.310	–	–	–	–
**DAP**	−0.001 (0.000)	**0.002**	−0.001 (0.001)	0.406	−0.000 (0.000)	0.307
**Total duration**	−0.108 (0.082)	0.191	–	–	–	–
**Fluoroscopy time**	−0.410 (0.209)	**0.053**	0.014 (0.288)	0.962	–	–

Post hoc univariate and multivariable analyses were performed to assess factors associated with the mental component summary (MCS). Variables with *p* < 0.05 in univariate analysis were included in the multivariable model. An additional multivariable model excluding fluoroscopy time was calculated due to its borderline significance in univariate analysis. β denotes the regression coefficient and SE the standard error. Bold values indicate statistical significance (*p* < 0.05). Abbreviations: MCS, mental component summary; SE, standard error; DAP, dose area product; BMI, body mass index.

**Table 3:** Post hoc univariate analysis of PCS adjusted for confounders.

Variable	Univariate Model β (SE)	*p*-Value
Age	−0.227 (0.081)	0.006
Sex	−2.908 (1.721)	0.094
Angiography before/after endovascular treatment	3.880 (2.622)	0.142
BMI	−0.236 (0.158)	0.140
DAP	−0.000 (0.000)	0.247
Total duration	−0.031 (0.073)	0.674
Fluoroscopy time	−0.271 (0.185)	0.147

Post hoc univariate analyses were performed to assess factors associated with the physical component summary (PCS). Only age showed a statistically significant association with PCS, while all other variables were not significant. Therefore, no multivariable model was performed. β denotes the regression coefficient and SE the standard error. Bold values indicate statistical significance (*p* < 0.05). Abbreviations: PCS, physical component summary; SE, standard error; DAP, dose area product; BMI, body mass index.

**Table 4:** Subscale-specific distribution of impairment within PCS and MCS (SF-36).

PCS ^1^	Relative Frequency	95% CI	MCS ^2^	Relative Frequency	95% CI
**Physical** **Functioning**	33.3%	7.5–70.1%	Emotional Role Limitation	40.0%	5.3–85.3%
**Physical Role** **Limitation**	55.6%	21.2–86.3%	Vitality	**80.0%**	28.4–99.5%
**Bodily Pain**	**88.9%**	51.8–99.7%	Mental Health	**80.0%**	28.4–99.5%
**General Health** **Perception**	55.6%	21.2–86.3%	Social Functioning	20.0%	0.5–71.6%

Relative frequencies of impairment in individual SF-36 subscales within ^1^ PCS (Physical Component Summary) (*n* = 9) and ^2^ MCS (Mental Component Summary) (*n* = 5), presented with 95% Clopper-Pearson exact confidence intervals (CI).

## Data Availability

The raw data supporting the conclusions of this article will be made available by the authors on request.

## Abbreviations

BMI	Body mass index
DAP	Dose area product
DSA	Digital subtraction angiography
EQ-5D	EuroQol 5-Dimension questionnaire
MCS	Mental Component Summary
PCS	Physical Component Summary
QoL	Quality of life
SD	Standard deviation
SF-12	12-Item Short Form Health Survey
SF-36	36-Item Short Form Health Survey

## References

[1] Lv X., Wu Z. (2025). World History of Neurointerventional Surgery.

[2] Behrens L., Adam A., Rubeck A., Schiele S., Müller G., Abrishami Y., Berlis A., Maurer C.J. (2024). Safety Aspects and Procedural Characteristics of Ambulatory Diagnostic Cerebral Catheter Angiography. Clin. Neuroradiol..

[3] Alakbarzade V., Pereira A.C. (2018). Cerebral catheter angiography and its complications. Pract. Neurol..

[4] Kobayashi Y., Yuzawa C., Hanaoka Y., Kobayashi K.-I., Kurashina M., Shimizu Y., Sato A., Sekijima Y. (2026). Technical Success of Stent Placement via Transradial Approach for Aberrant Right Subclavian Artery Stenosis. Vasc. Endovasc. Surg..

[5] Khan I., Johnson H.E., Drewes N.B., Traylor B.A., Catalano A.F., Delfino K., Dayoub H. (2025). 3D Geometric Analysis of Anterior Circulation Anatomy in Patients with Intracranial Aneurysms. World Neurosurg..

[6] Padhi R., Shethna V.S., Dhanasekaran J., Kocer N., Rao M., Shetty K.K. (2025). Direct Carotid Puncture Mechanical Thrombectomy in Medium Vessel Occlusion (MEVO) Stroke Using Obtura Closure Device for Hemostasis. Vasc. Endovasc. Surg..

[7] Choudhri O., Schoen M., Mantha A., Feroze A., Ali R., Lawton M.T., Do H.M. (2016). Increased risk for complications following diagnostic cerebral angiography in older patients: Trends from the Nationwide Inpatient Sample (1999–2009). J. Clin. Neurosci..

[8] Jolly S.S., Yusuf S., Cairns J., Niemelä K., Xavier D., Widimsky P., Budaj A., Niemelä M., Valentin V., Lewis B.S. (2011). Radial versus femoral access for coronary angiography and intervention in patients with acute coronary syndromes (RIVAL): A randomised, parallel group, multicentre trial. Lancet.

[9] Windecker S., Kolh P., Alfonso F., Collet J.-P.P., Cremer J., Falk V., Filippatos G., Hamm C., Head S.J., Jüni P. (2014). 2014 ESC/EACTS Guidelines on myocardial revascularization: The Task Force on Myocardial Revascularization of the European Society of Cardiology (ESC) and the European Association for Cardio-Thoracic Surgery (EACTS) Developed with the special contribution of the European Association of Percutaneous Cardiovascular Interventions (EAPCI). Eur. Heart J..

[10] Valgimigli M., Gagnor A., Calabró P., Frigoli E., Leonardi S., Zaro T., Rubartelli P., Briguori C., Andò G., Repetto A. (2015). Radial versus femoral access in patients with acute coronary syndromes undergoing invasive management: A randomised multicentre trial. Lancet.

[11] Elfil M., Ghaith H.S., Jain A., Spirollari E., Sacknovitz A., Elmashad A., Aladawi M., Salem M.M., Najdawi Z., El-Ghanem M. (2025). Transradial Access as an Innovative Approach for Endovascular Thrombectomy: A Living Systematic Review and Meta-Analysis. Cardiol. Rev..

[12] Hamouda A.M., El Gazar T., Ali M.A., Jha S.K., Cwajna M., Kendall N., Derhab M., Ghozy S., Pennington Z., Kumar R. (2025). Transradial versus transfemoral access in diagnostic cerebral angiography: A comprehensive systematic review and meta-analysis of clinical outcomes and complications. Neuroradiology.

[13] Hoffman H., Jalal M.S., Masoud H.E., Pons R.B., Rodriguez Caamaño I., Khandelwal P., Prakash T., Gould G.C. (2021). Distal Transradial Access for Diagnostic Cerebral Angiography and Neurointervention: Systematic Review and Meta-analysis. Am. J. Neuroradiol..

[14] Khanna O., Sweid A., Mouchtouris N., Shivashankar K., Xu V., Velagapudi L., Stricsek G., Amllay A., Texakalidis P., Gooch M.R. (2019). Radial Artery Catheterization for Neuroendovascular Procedures. Stroke.

[15] Kok M.M., Weernink M.G.M., von Birgelen C., Fens A., van der Heijden L.C., van Til J.A. (2018). Patient preference for radial versus femoral vascular access for elective coronary procedures: The PREVAS study. Catheter. Cardiovasc. Interv..

[16] Satti S.R., Vance A.Z., Golwala S.N., Eden T. (2017). Patient Preference for Transradial Access over Transfemoral Access for Cerebrovascular Procedures. J. Vasc. Interv. Neurol..

[17] Brunet M.-C., Chen S.H., Peterson E.C. (2020). Transradial access for neurointerventions: Management of access challenges and complications. J. Neurointerv. Surg..

[18] Stone J.G., Zussman B.M., Tonetti D.A., Brown M., Desai S.M., Gross B.A., Jadhav A., Jovin T.G., Jankowitz B. (2020). Transradial versus transfemoral approaches for diagnostic cerebral angiography: A prospective, single-center, non-inferiority comparative effectiveness study. J. Neurointerv. Surg..

[19] Atallah E., El Naamani K., Momin A.A., Abbas R., Jain P., Hunt A., Sambangi A., Carreras A., El Fadel O., Gooch M.R. (2024). Transradial versus transfemoral access routes for diagnostic cerebral angiography: A large single-center comparative cost-analysis study. J. Neurosurg..

[20] Rosskopf J., Kifmann J., Schmitz B., Braun M. (2025). Quality of Life After Transradial Access in Cerebral Angiography: A SF-12 Analysis Using a Then-Test Design. Healthcare.

[21] Wang W., Ma Y., Wang C., Shi P., Lv W., Fan G., Sun C. (2024). Risk factors for puncture-related complications after cerebrovascular angiography and neuroendovascular intervention with distal transradial approach. BMC Neurol..

[22] Ma P., Gong Z., Du M., Zhu D., Li P., Fang Y. (2024). Mediastinal hematoma after trans-radial cerebral angiography: A case report. BMC Neurol..

[23] Tso M.K., Rajah G.B., Dossani R.H., Meyer M.J., McPheeters M.J., Vakharia K., Waqas M., Snyder K.V., Levy E.I., Siddiqui A.H. (2022). Learning curves for transradial access versus transfemoral access in diagnostic cerebral angiography: A case series. J. Neurointerv. Surg..

[24] Zwaan E.M., Cheung E.S., Ijsselmuiden A.J.J.J., Holtzer C.A.J.J., Schreuders T.A.R.R., Kofflard M.J.M.M., Coert J.H. (2022). Upper Extremity Function following Transradial Percutaneous Coronary Intervention: Results of the ARCUS Trial. J. Interv. Cardiol..

[25] Ayyaz Ul Haq M., Rashid M., Gilchrist I.C., Bertrand O., Kwok C.S., Wong C.W., Mansour H.M., Baghdaddy Y., Nolan J., van Leeuwen M.A.H. (2018). Incidence and clinical course of limb dysfunction post cardiac catheterization: A systematic review. Circ. J..

[26] Van Leeuwen M.A.H., Van Der Heijden D.J., Hermie J., Lenzen M.J., Selles R.W., Ritt M.J.P.F., Kiemeneij F., Zijlstra F., Van Mieghem N.M., Van Royen N. (2017). The long-term effect of transradial coronary catheterisation on upper limb function. EuroIntervention.

[27] Kifmann J., Braun M., Steinhart J., Sollmann N., Kloth C., Pedro M., Dreyhaupt J., Beer M., Schmitz B., Rosskopf J. (2026). Effect of the Transradial Approach on Wrist Function in Diagnostic Cerebral Angiography. Healthcare.

[28] Bullinger M. (1996). Assessment of health related quality of life with the SF-36 Health Survey. Rehabilitation.

[29] Ellert U., Kurth B.M. (2013). Gesundheitsbezogene Lebensqualität bei Erwachsenen in Deutschland: Ergebnisse der Studie zur Gesundheit Erwachsener in Deutschland (DEGS1). Bundesgesundheitsblatt-Gesundheitsforschung-Gesundheitsschutz.

[30] Reder S.R., Hardt J., Brockmann M.A., Brockmann C., Kim S., Kawulycz M., Schulz M., Kantelhardt S.R., Petrowski K., Fischbeck S. (2025). Profiling disease experience in patients living with brain aneurysms by analyzing multimodal clinical data and quality of life measures. Sci. Rep..

[31] Ware J.J., Kosinski M., Keller S.D. (1996). A 12-Item Short-Form Health Survey: Construction of scales and preliminary tests of reliability and validity. Med. Care.

[32] Drixler K., Morfeld M., Glaesmer H., Brähler E., Wirtz M.A. (2020). Validation of the Short-Form-Health-Survey-12 (SF-12 Version 2.0) assessing health-related quality of life in a normative German sample. Z. Psychosom. Med. Psychother..

[33] Lin Y., Yu Y., Zeng J., Zhao X., Wan C. (2020). Comparing the reliability and validity of the SF-36 and SF-12 in measuring quality of life among adolescents in China: A large sample cross-sectional study. Health Qual. Life Outcomes.

[34] Ara R., Brazier J. (2008). Deriving an algorithm to convert the eight mean SF-36 dimension scores into a mean EQ-5D preference-based score from published studies (where patient level data are not available). Value Health.

[35] Batóg P., Rencz F., Péntek M., Gulácsi L., Filipiak K.J., Prevolnik Rupel V., Simon J., Brodszky V., Baji P., Závada J. (2018). EQ-5D studies in cardiovascular diseases in eight Central and Eastern European countries: A systematic review of the literature. Kardiol. Pol..

[36] Rabin R., de Charro F. (2001). EQ-5D: A measure of health status from the EuroQol Group. Ann. Med..

[37] Jenkinson C., Layte R., Jenkinson D., Lawrence K., Petersen S., Paice C., Stradling J. (1997). A shorter form health survey: Can the SF-12 replicate results from the SF-36 in longitudinal studies?. J. Public Health Med..

[38] Riddle D.L., Lee K.T., Stratford P.W. (2001). Use of SF-36 and SF-12 health status measures: A quantitative comparison for groups versus individual patients. Med. Care.

[39] Bushnik T., Kreutzer J.S., DeLuca J., Caplan B. (2018). SF-36/SF-12. Encyclopedia of Clinical Neuropsychology.

[40] Pickard A.S., Johnson J.A., Penn A., Lau F., Noseworthy T. (1999). Replicability of SF-36 summary scores by the SF-12 in stroke patients. Stroke.

[41] Braun M., Kifmann J., Steinhart J., Sollmann N., Kloth C., Pedro M., Hlavac M., Dreyhaupt J., Beer M., Schmitz B. (2026). Prospective Follow-Up Assessment of Wrist Function After the Transradial Approach for Diagnostic Cerebral Catheter Angiography. J. Clin. Med..

[42] Morfeld M., Bullinger M. (2008). Der SF-36 Health Survey zur erhebung und dokumentation gesundheitsbezogener lebensqualität. Phys. Med. Rehabil. Kurortmed..

[43] Ware J.E.J., Sherbourne C.D. (1992). The MOS 36-item short-form health survey (SF-36). I. Conceptual framework and item selection. Med. Care.

[44] Jenkinson C. (1998). The SF-36 physical and mental health summary measures: An example of how to interpret scores. J. Health Serv. Res. Policy.

[45] McHorney C.A., Ware J.E.J., Raczek A.E. (1993). The MOS 36-Item Short-Form Health Survey (SF-36): II. Psychometric and clinical tests of validity in measuring physical and mental health constructs. Med. Care.

[46] Norman G.R., Sloan J.A., Wyrwich K.W. (2003). Interpretation of changes in health-related quality of life: The remarkable universality of half a standard deviation. Med. Care.

[47] Pala A., Pawlikowski A., Brand C., Schmitz B., Wirtz C.R., König R., Kapapa T. (2019). Quality of Life After Treatment of Unruptured Intracranial Aneurysms. World Neurosurg..

[48] Dammann P., Wittek P., Oppong M.D., Hütter B., Jabbarli R., Wrede K., Wanke I., Mönninghoff C., Kaier K., Frank B. (2019). Relative health-related quality of life after treatment of unruptured intracranial aneurysms: Long-term outcomes and influencing factors. Ther. Adv. Neurol. Disord..

[49] Dandurand C., Zhou L., Fitzmaurice G., Prakash S., Redekop G., Haw C., Gooderham P. (2021). Quality of life scores in patients with unruptured cerebral aneurysm: Prospective cohort study. J. Clin. Neurosci..

[50] Cooper C.J., El-Shiekh R.A., Cohen D.J., Blaesing L., Burket M.W., Basu A., Moore J.A. (1999). Effect of transradial access on quality of life and cost of cardiac catheterization: A randomized comparison. Am. Heart J..

[51] Reddy B.K., Brewster P.S., Walsh T., Burket M.W., Thomas W.J., Cooper C.J. (2004). Randomized comparison of rapid ambulation using radial, 4 French femoral access, or femoral access with AngioSeal closure. Catheter. Cardiovasc. Interv..

[52] Koltowski L., Koltowska-Haggstrom M., Filipiak K.J., Kochman J., Golicki D., Pietrasik A., Huczek Z., Balsam P., Ścibisz A., Opolski G. (2014). Quality of life in patients with ST-segment elevation myocardial infarction undergoing percutaneous coronary intervention-Radial versus femoral access (from the OCEAN RACE trial). Am. J. Cardiol..

[53] Sciahbasi A., Fischetti D., Picciolo A., Patrizi R., Sperduti I., Colonna G., Summaria F., Montinaro A., Lioy E. (2009). Transradial access compared with femoral puncture closure devices in percutaneous coronary procedures. Int. J. Cardiol..

[54] Sandoval Y., Bell M.R., Gulati R. (2019). Transradial Artery Access Complications. Circ. Cardiovasc. Interv..

[55] Roczniak J., Koziołek W., Piechocki M., Tokarek T., Surdacki A., Bartuś S., Chyrchel M. (2021). Comparison of Access Site-Related Complications and Quality of Life in Patients after Invasive Cardiology Procedures According to the Use of Radial, Femoral, or Brachial Approach. Int. J. Environ. Res. Public Health.

[56] Rossmann T., Pruidze P., Mayerhofer J., Veldeman M., Pfisterer W.K., Weninger W.J., Meng S. (2026). Radial Artery Occlusion Impairs Median Nerve Perfusion—A Study Using Microvascular Imaging in Healthy Volunteers. Diagnostics.

[57] Sun Z., Wei W., Jia C., Zhang H., Zhou C., Luo G., Li W., Li S., Wang Y., Guo S. (2025). The value of ultrasound in transradial access cerebral angiography. Front. Neurol..

[58] Peng T., Tian H., Zhu B., Liu J., Zhang L., Xie Y., Dan B. (2025). A transradial approach directly using a distal access catheter for symptomatic intracranial vertebral artery and basilar artery stenosis: A single-center study. Clin. Neurol. Neurosurg..

[59] Jiang S., Yu S., Gu B., Li X., Xiao H., Zhao D., Ma X. (2025). Distal versus proximal transradial access for diagnostic cerebral angiography: A single-center experience. J. Clin. Neurosci..

[60] Orscelik A., Senol Y.C., Kobeissi H., Ghozy S., Bilgin C., Arul S., Kadirvel R., Brinjikji W., Kallmes D.F. (2023). Distal versus conventional transradial access for diagnostic cerebral angiography and neurointerventional procedures: A systematic review and meta-analysis. Interv. Neuroradiol..

[61] Gómez-Pena S., Trejo C., Pérez-García C., López-Frías A., Rosati S., Schmolling Á.H., Moreu M. (2025). Transulnar approach as an alternative access site for neuroendovascular procedures. Neuroradiol. J..

[62] Wang W., Ma Y., Fan G., Shi P., Yang M., Lv W., Wang C. (2025). Comparative analysis of cerebral angiography via right versus left radial artery approaches. BMC Neurol..

[63] Liu C., Deng G., Wang Y., Ao D., Huang H., Zhang Y., Xie Y., Yu Y., Kong Q., Li G. (2026). Efficacy and safety of heparin in preventing embolic events during transradial cerebral angiography: A randomised controlled trial. Stroke Vasc. Neurol..

[64] He R., Zhang X., Yan L., Tao M., Wang T., Zhong C., Xiao K., Gan L., Luo M. (2025). Low-dose heparin reduces radial artery injury in transradial cerebral angiography: A 375-case retrospective study. Neurol. Res..

[65] Pan D., Yang J., Liu M., Xu Y., Feng J., Li H., Luo W., He B., Xiao S., Yang X. (2025). Efficacy of Papaverine to Prevent Radial Artery Spasm During Transradial Cerebral Angiography (PASS): Rationale and design. Stroke Vasc. Neurol..

